# Waterpipe smoking in Saudi Arabia: Action plan

**DOI:** 10.18332/tid/108663

**Published:** 2019-04-24

**Authors:** Naif Alanazi

**Affiliations:** 1 Department of Public health, College of Health Sciences, Saudi Electronic University, Riyadh, Saudi Arabia

**Keywords:** smoking, tobacco smoking, waterpipe smoking, hookah

**Dear Editor,**

Waterpipe use has grown exponentially during the last 20 years in Middle-East and Western countries, including the United States. In Saudi Arabia there has been an increasing trend in waterpipe smoking among teenagers and young adults^1,2^. In addition to the spread of waterpipe lounges and cafés, some individuals use private places, such as *Estrahas*, where they can gather for talking and smoking. Finding an *Estraha* without a waterpipe smoker is uncommon. Some *Estrahas* are explicitly used to gather with friends to play cards, play video games, and smoke waterpipe.

Epidemiological data of waterpipe smoking in Saudi Arabia have shown alarming evidence of high waterpipe usage among teenagers and college students, even though studies have overall shown a considerable variation in the prevalence of waterpipe use. For instance, a study of male medical college students in the eastern region of the country measured that the prevalence of waterpipe smoking was 12.6%^3^. Another study conducted in Al-Hassa, also in eastern Saudi Arabia, showed that current tobacco use was 30.3% among high school students, and among those current tobacco smokers, 53.9% were also waterpipe users^4^. Conducted in some colleges of Qassim University, another study revealed that the total prevalence of waterpipe use was 40%^5^. Moreover, a recent national survey indicated that the overall prevalence of current tobacco smoking was 12.2% among those 15 years or older with 4.3% of the total population daily waterpipe users and 1.4% current daily smokers of cigarettes/cigar and waterpipe users^6^.

Regardless of the variation of waterpipe smoking prevalence, the available statistics are presenting waterpipe use as a severe public health issue in Saudi Arabia. To counterbalance waterpipe smoking in Saudi Arabia, we should consider the following action plan:

First, we need to identify the magnitude of these smoking phenomena. Better social and epidemiological assessments of waterpipe smoking are needed to objectively define the problem and its scope in terms of popularity and its negative consequences on health and society.

Second, we need to create educational interventions. We should identify factors that have the potential to influence waterpipe smoking behaviour. The factors may include health knowledge, personal attitude and perceptions, misconceptions, and beliefs toward waterpipe smoking. We should identify the factors that either encourage or discourage waterpipe use and develop appropriate prevention/intervention programs targeting waterpipe smoking behaviour.

Third, we need policy assessments, implementation, and improved regulatory actions that require health policymakers to review, edit and update current smoking policies in Saudi Arabia, and ensure regulations are enforced. Such policies should include age restriction regulations for entering waterpipe lounges/cafés, the prohibition of smoking in public places or selling tobacco to minors, appropriate monitoring of the constituents of waterpipe tobacco, the standardization of waterpipe tobacco production, the addition of visible warning labels in each waterpipe tobacco box, and the removal of deceptive labels or descriptions, such as ‘0% tar’, ‘0% nicotine’, or ‘100% natural’, etc.

To conclude, health officials and public health professionals should declare waterpipe use a severe threat to public health. More funds and research are required to build evidence-based comprehensive tobacco waterpipe prevention/intervention strategies. Available facts and evidence can be used to begin policy and regulatory actions. Delaying or taking no action will lead to continued ineffective control over waterpipe use that may result in adverse public health outcomes.

## CONFLICTS OF INTEREST

The author has completed and submitted the ICMJE Form for Disclosure of Potential Conflicts of Interest and none was reported.
